# Isolation of Hox Cluster Genes from Insects Reveals an Accelerated Sequence Evolution Rate

**DOI:** 10.1371/journal.pone.0034682

**Published:** 2012-06-07

**Authors:** Heike Hadrys, Sabrina Simon, Barbara Kaune, Oliver Schmitt, Anja Schöner, Wolfgang Jakob, Bernd Schierwater

**Affiliations:** 1 ITZ, Division of Ecology and Evolution, Stiftung Tieraerztliche Hochschule Hannover, Hannover, Germany; 2 Dept. of Ecology and Evolutionary Biology, Yale University, New Haven, Connecticut, United States of America; 3 American Museum of Natural History, New York City, New York, United States of America; American Museum of Natural History, United States of America

## Abstract

Among gene families it is the Hox genes and among metazoan animals it is the insects (Hexapoda) that have attracted particular attention for studying the evolution of development. Surprisingly though, no Hox genes have been isolated from 26 out of 35 insect orders yet, and the existing sequences derive mainly from only two orders (61% from Hymenoptera and 22% from Diptera). We have designed insect specific primers and isolated 37 new partial homeobox sequences of Hox cluster genes *(lab, pb, Hox3, ftz, Antp, Scr, abd-a, Abd-B, Dfd, and Ubx)* from six insect orders, which are crucial to insect phylogenetics. These new gene sequences provide a first step towards comparative Hox gene studies in insects. Furthermore, comparative distance analyses of homeobox sequences reveal a correlation between gene divergence rate and species radiation success with insects showing the highest rate of homeobox sequence evolution.

## Introduction

*Antp*-class genes code for homeodomain-containing transcription factors that function in cell fate determination and embryonic development [1], [2]–[4]. In Bilateria up to 100 *Antp*-class genes (including paralogs) can be divided into 30 gene families belonging to four major groups: HOX/PARAHOX genes (45 genes, four gene families), HOX-related genes (nine genes, five gene families), NK genes (16 genes, seven gene families), and NK-related genes (28 genes, 18 pseudogenes, 14 gene families). From the simplest Bilateria, the Platyhelmintha, 15 *Antp*-class genes are known and from the Arthropoda 37 (34 in Insecta). These genes have been of outstanding importance for metazoan radiation and provided deep insights into both, the phylogenetic patterns and the genetic mechanisms of animal bauplan development [5], [6]–[11]. Particularly Hox genes have attracted much attention since they define the identities of bauplan units (e.g. segments) along the anterior-posterior axis of the embryo [5], [12], [13]. Hox genes have been known from all Bilateria and Hox-like genes also from diploblastic metazoans, including Placozoa and Cnidaria [14], [15], [16].

Despite the importance of insects as the largest animal group on earth, and Hox genes as the most influential gene class in EvoDevo research, Hox genes have been isolated from only 8 out of some 35 insect orders yet. The full repertoire of Antennapedia genes has so far only been reported for *Folsomia candida*, *Tribolium castaneum* and *Drosophila melanogaster*. The majority of all sequences derive from two orders only, the Hymenoptera and the Diptera. In *Drosophila melanogaster* the Hox-Cluster is organized in two separate units: (a) the Antennapedia complex consisting of the Hox genes *labial* (*lab*), *proboscipedia* (*pb*), *Hox3 (z2, zen, bcd)*, *fushi tarazu* (*ftz*), *Deformed* (*Dfd*), *Sex combs reduced* (*Scr*) and *Antennapedia* (*Antp*), and (b) the Bithorax complex which includes *Ultrabithorax* (*Ubx*), *abdominal-A* (*abd-A*) and *Abdominal-B* (*abd-B*) [17], [18], [19]. This split is likely an aut-apomorphy of the Diptera since all of the above mentioned genes may be linked in a single cluster in other insects, e.g. Coleoptera [8], [20]–[24].

It is highly unfortunate that very little is known about *Antp* genes in basal insects and that the origin and radiation of Hox genes in insects remains widely unresolved. Marden et al. [25] highlight the crucial importance of isolating Hox genes particularly from basal Pterygota in order to reveal intermediate stages of evolution of appendages and shed some light on the early evolution of flying insects. We here report on the successful isolation of 37 new homeobox fragments from six insect orders of crucial phylogenetic position, the apterygote Diplura and Archaeognatha, and the pterygote orders Ephemeroptera, Odonata, Plecoptera, and Dermaptera. We furthermore show that the rate of homeobox sequence evolution in the fastest radiating animal group, the insects, has been faster than in non-insects.

## Materials and Methods

### Animal Material and DNA Extraction

Specimens of *Campodea fragilis* (Diplura) and *Lepismachilis y-signata* (Archaeognatha) were kindly supplied by Karen Meusemann (ZFMK Bonn, Germany). *Sympetrum sanguineum, Ischnura elegans* (both Odonata) and *Baetis sp.* (Ephemeroptera) were collected at a small pond close to our institute in Hannover. The *Nemoura cinerea* (Plecoptera) sample was kindly supplied by the National Museum Prague (Czechia) and *Forficula auricularia* (Dermaptera) was found in Hannover in a private garden. Tissue samples (legs of *S. sanguineum* or else whole animals) were preserved in ethanol (80%) and stored at 4°C. Whole genomic DNA was extracted according to Hadrys et al. [26], [27]. (No specific permits were required for the described field studies. The locations are not privately-owned or protected in any way and the field studies did not involve endangered or protected species.).

### PCR Amplification

Partial homeobox sequences of the genes *Deformed* (*Dfd*), *Sex combs reduced* (*Scr*), *Ultrabithorax* (*Ubx*) and *abdominal-A* (*abd-A*) were amplified by PCR with degenerate primers. We designed “insect specific” degenerate primers, which specifically amplify partial homeobox sequences of between 120 and 164 bp of the target genes (Table 1). In addition, homeobox sequences were amplified by various combinations of four degenerated forward primers and five degenerated reverse primers reported in Cook et al. [28].

**Table 1:** New “insect specific” degenerate primers designed for the amplification of large homeobox fragments of *Dfd, Scr, Ubx,* and *abd-A* Hox genes.

Name	Sequence (5′ –3′)	AT (°C)	Fragment (bp)
Dfd1fw	CAAGCGGCAGCGGACNCSNTAYAC	58	160
Dfd1rev	TCTTCCTCCGCACGTTCTTNGTRTTNGG	57	
Scr1fw	GCAGCGGACCTCCTACACCMGNTAYCARAC	62	128
Scr1rev	TCATGGTGGCCATCTTGTGYTCYTTYTTCC	57	
Ubx3fw	GCCGGCAGACCTACACCMGNTAYCARAC	61	145
Ubx3rev	CTCCTGCTCGTTCAGCTCYTTDATNGC	57	
abdAfw	CGGCGGCGGGGNMGNCARAC	59	164
abdArev	GGGCCTGCTCGTTGATCTCYTTNACNGC	60	

Given are the primer sequences (forward = fw, reverse = rev), optimal annealing temperatures (AT) and expected fragment length of PCR products.

#### “Insect specific” degenerate Primer PCR

Reactions were carried out in a total volume of 30 µl containing 40 pmol of each primer pair, 3.3 mmol of dNTP mix, and 1.5 U of Taq-Polymerase (Invitrogen). PCR started with an initial denaturation (93°C for 2 min) followed by 45 amplification cycles: denaturing at 92°C for 30 sec, annealing at 55 to 75°C (optimized for each primer pair and organism) for 35 sec, elongation at 72°C for 30 sec. All PCRs finished with a final elongation at 72°C for 5 min. PCR products were purified with Montage PCR Centrifugal Filter Devices (Millipore).

#### Degenerate Primer PCR [28]

The 50 ml reaction mix contained: 1× amplification buffer, 4 mM MgCl_2_, 0.2 mM dNTPs, 10 pM each primer and 0.04 U Taq DNA polymerase (Bioline). The ramp up PCR started with an initial denaturation (95°C for 5 min) followed by 6 amplification cycles: denaturing at 94°C for 45 sec, annealing started at 48°C for 10 sec followed by a ramp to 56°C (0.1°C/sec) and a ramp to 72°C (0.2°C/sec), elongation at 72°C for 10 sec, and subsequent 30 amplification cycles: denaturing at 94°C for 30 sec, annealing started at 53°C for 10 sec followed by a ramp to 62°C (0.1°C/sec), elongation at 72°C for 30 sec and finished with a final elongation at 72°C for 5 min. PCR products of the expected length (∼70 – ∼100 bp) were cut out of the gel and purified through ethanol precipitation.

### Cloning and Sequencing

The purified products were A-tailed and inserted into the pGEM-T plasmid vector (Promega) and cloned into *E. coli* (Invitrogen) following the manufacturer’s instructions. Clones were sequenced in both directions on an ABI PRISM 310 Genetic Analyzer (Applied Biosystems) using BigDye® Terminator Cycle Sequencing Kit (v.1.1, Applied Biosystems). Sequences were analyzed and aligned using SeqMan II 5.03 (DNAStar, Lasergene) and ClustalW [29].

### Calculating Divergence Rates

To infer rates of molecular evolution of insect Hox genes *p*-distances within groups were calculated using MEGA5 [30]. These divergence rates of insect Hox genes were compared to calculated divergence rates of other arthropod classes and Mammalia (see Table S1 for their GenBank accession numbers). Information from fossil records was used to estimate the absolute rates (in % per million years) at which the different lineages have accumulated mutations in their homeobox sequences.

## Results and Discussion

**Figure 1:**
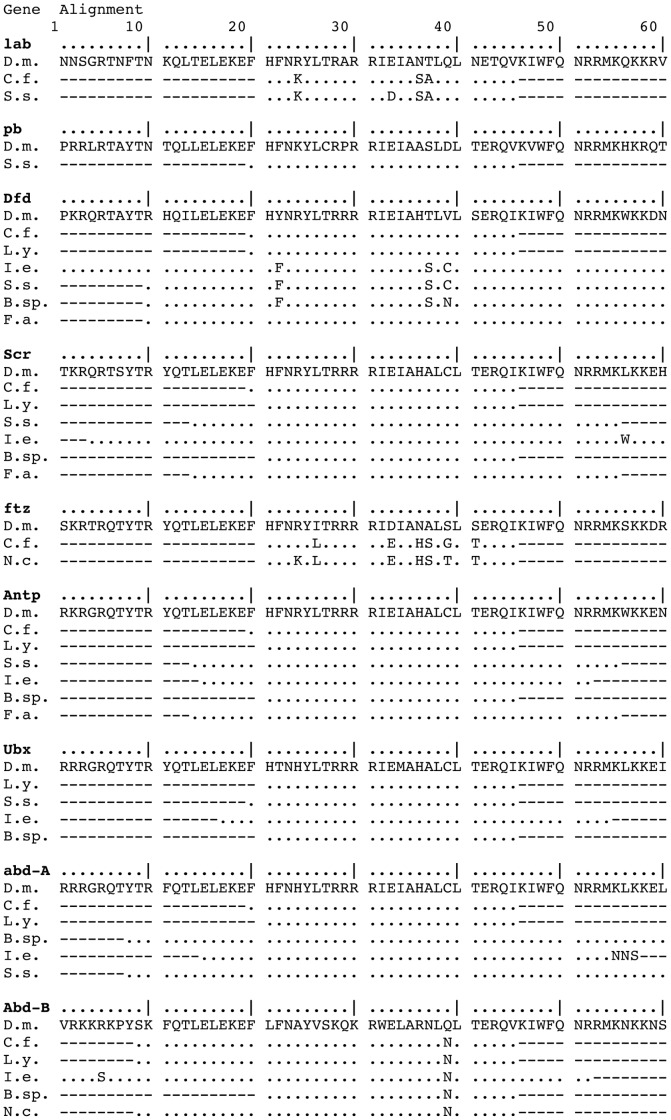
Alignment of 37 new hexapod Hox gene homeodomains. The newly isolated sequences of *lab, pb, Dfd, Scr, ftz, Antp, Ubx, abd-A* and *abd-B* from the dipluran *Campodea fragilis* (C.f.), the archaeognath *Lepismachilis y-signata* (L.y.), the odonates *Ischnura elegans* (I.e.) and *Sympetrum sanguineum* (S.s.), the ephemeropteran *Baetis* sp. (B.sp.), the plecopteran *Nemoura cinerea* (N.c.) and the dermapteran *Forficula auricularia* (F.a.) are aligned to their *Drosophila melanogaster* (D.m.) homolog. Dots indicate identical position.

In this study we have isolated the first homeobox sequences of Hox cluster genes from six insect orders: Diplura (*lab, Dfd, Scr, Antp, ftz, abd-A, Abd-B*), Archaeognatha (*Dfd*, *Scr, Antp, Ubx, abd-A, Abd-B*), Ephemeroptera (*Dfd*, *Scr, Antp*, *Ubx*, *abd-A, Abd-B*), Odonata (*lab, pb, Hox3, Dfd*, *Scr, Antp*, *Ubx*, *abd-A, Abd-B*), Plecoptera (*Dfd*, *Scr, Antp*, *ftz, Ubx, Abd-B*), and Dermaptera (*Dfd*, *Scr*) (amino acid alignments are shown in Fig. 1). These 37 new sequences (Table S1) fill in crucial gaps both at the base of insects as well as at the base of Pterygota (Table 2). The new data raise the number of insect orders with reported Hox cluster gene sequences from 8 to 14 and the number of known gene sequences in the matrix from 67 to 101. In these numbers we include sequences from the 8 Hox genes (*lab*, *pb*, *Dfd*, *Scr*, *Antp*, *Ubx*, *abd-A*, *abd-B*) as well as from the two homeotic genes, *Hox3* (*bicoid*) and *ftz*, which are integrated in the insect Hox cluster (or clusters in the case of Diptera).

**Table 2:** Hox genes known from the different insect orders.

Order (Infraclass)	*lab*	*pb*	*Hox3*	*Dfd*	*Scr*	*ftz*	*Antp*	*Ubx*	*abd-A*	*Abd-B*
Diplura	***here***	*–*	*–*	***here***	***here***	***here***	***here***	*–*	***here***	***here***
Collembola	*1*	*1*	*1*	*1*	*1*	*1*	*1*	*1*	*1*	*1*
Protura	*–*	*–*	*–*	*–*	*–*	*–*	*–*	*–*	*–*	*–*
Archaeognatha	*–*	*–*	*–*	***here***	***here***	*1*	***here***	***here***	***here***	***here***
Thysanura	*1*	*1*	*1*	*1*	*1*	*1*	*1*	*–*	*1*	*1*
Ephemeroptera	*–*	*–*	*–*	***here***	***here***	*–*	***here***	***here***	***here***	***here***
Odonata	***here***	***here***	***here***	***here***	***here***	*–*	***here***	***here***	***here***	***here***
Plecoptera	*–*	*–*	*–*	***here***	***here***	***here***	***here***	***here***	*–*	***here***
Dermaptera	*–*	*–*	*–*	***here***	***here***	*1*	***here***	*–*	*–*	*–*
Orthoptera	*1*	*–*	*1*	*1*	*2*	*–*	*2*	*2*	*2*	*1*
Blattodea	*–*	*–*	*–*	*–*	*1*	*–*	*–*	*–*	*–*	*–*
Hemiptera	*1*	*26*	*1*	*1*	*34*	*–*	*2*	*3*	*31*	*1*
Hymenoptera	*2*	*3*	*1*	*1*	*2*	*2*	*2*	*>100*	*>100*	*4*
Diptera	*8*	*4*	*4*	*9*	*9*	*13*	*6*	*14*	*9*	*8*
Coleoptera	*1*	*1*	*1*	*1*	*1*	*1*	*1*	*1*	*1*	*1*
Lepidoptera	*1*	*1*	*1*	*2*	*2*	*-*	*3*	*5*	*3*	*1*
Embioptera, Notoptera, Mantodea, Mantophasmatodea, Isoptera, Phasmatodea,Zoraptera, Psocoptera, Phthiraptera,Thysanoptera, (Hemimetabola) Megaloptera,Raphidioptera, Neuroptera, Trichoptera,Mecoptera, Siphonaptera, Strepsiptera (Holometabola)	No data available					No data available				

* an unambiguous distinction between *Scr* and *Antp* based on short sequence fragment is not possible (see text).

The full complement of Hox cluster genes has so far been known from Collembola, Diptera, and Coleoptera only. Partial information now includes 15 insect orders, and no information is available from at least 19 orders. Here  =  this study.

### Homology Assignment and Database Extension

**Figure 2:**
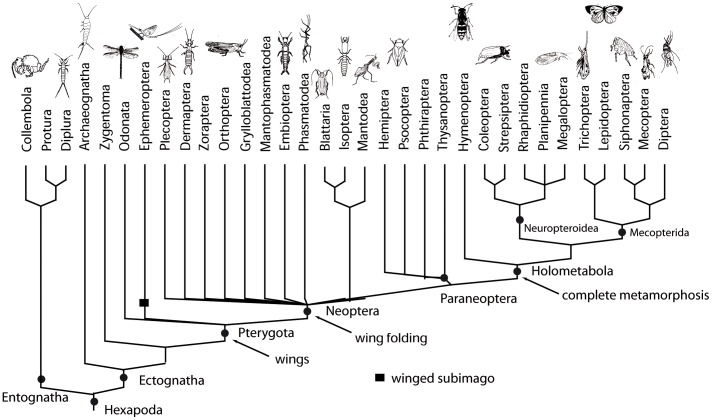
Neighbor-Joining tree of the 25 new Hox gene sequences (*lab, pb, Dfd*, *Ubx, abd-A, Abd-B*) and known orthologs from other insects (GenBank accession numbers: *Folsomia candida* AF361326, AF361327, AF361329, AF361333, AF361334, AF361335; *Drosophila melanogaster*; NM_057265, X63728, X05136, X76210, X54453, X16134; *Tribolium castaneum* AF231104, AF187068, U81039, AF146649, AF017415, AF227923). Sequences from this study are in bold. Note that all new sequences group to expected homologs.

**Figure 3:**
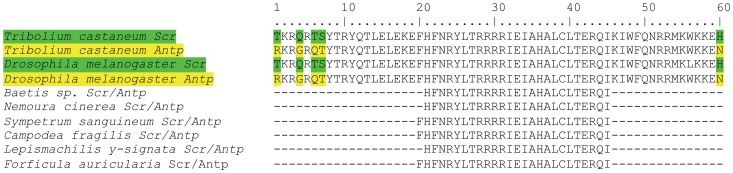
Alignment of Scr and Antp homeodomain fragments. Shown is the alignment to *Tribolium castaneum* (AF228509, AF227628) and *Drosophila melanogaster* (M20705, X05228) sequences. The six new and short *Scr* and *Antp* fragments differ in their homeobox sequence but are identical at the amino acid level. Amino acid substitutions between *Scr* and *Antp* are shown in green and yellow, respectively.

Homolog identification of the isolated Hox genes is widely, but not completely non-problematic. All assignments shown in Table 2 are the immediate assignments according to BLAST searches. As it has been shown that caution should be taken when using the best BLAST hit to infer gene homology [31]–[33], we performed phylogenetic analyses to further test the assignments of the newly isolated Hox genes. In these analyses (Neighbor-Joining, NJ) with published homeobox sequences the new homeobox sequences for *lab, pb, Dfd, Ubx, abd-A, Abd-B* group into the expected clades. The genes *Scr, ftz,* and *Antp* are generally problematic. Even their full length homeodomain sequences do not allow an unambiguous assignment in a standard distance analysis (Fig. S1). A Neighbour Joining analysis of only the six potentially unambiguous new homeobox sequences (*lab, pb, Dfd, Ubx, Abd-B*) groups all new fragments into the expected clades of homologs from other insects and thus confirms the results of NCBI Blast (Fig. 2), except for *abd-A* which appears paraphyletic. Based on the partial homeobox sequences we cannot unambiguously distinguish between *Scr* and *Antp* homeobox fragments in Diplura, Archaeognatha, Ephemeroptera, Odonata, Plecoptera and Dermaptera. The isolated homeobox fragments differ between orders, but no amino acid substitutions are found in the short fragment spanning homeodomain positions 20 to 45 (Fig. 3). For these gene fragments more sequence information is required to distinguish between the two alternatives, since amino acid substitutions have been known to occur at positions 1, 4, 6, 7 and 60 only (Fig. 3). We believe that we have amplified both genes (different homeobox sequences) but we are reluctant to suggest an assignment to the *Scr* or *Antp* gene family in the absence of unambiguous differences in the homeodomain. For odonates we have verified the correct assignment of the new gene fragments to their *Scr*, *Antp*, and *Ubx* gene families also by RACE-PCR, amplifying full length homeobox sequences for developmental studies (data will be reported elsewhere).

**Figure 4:**
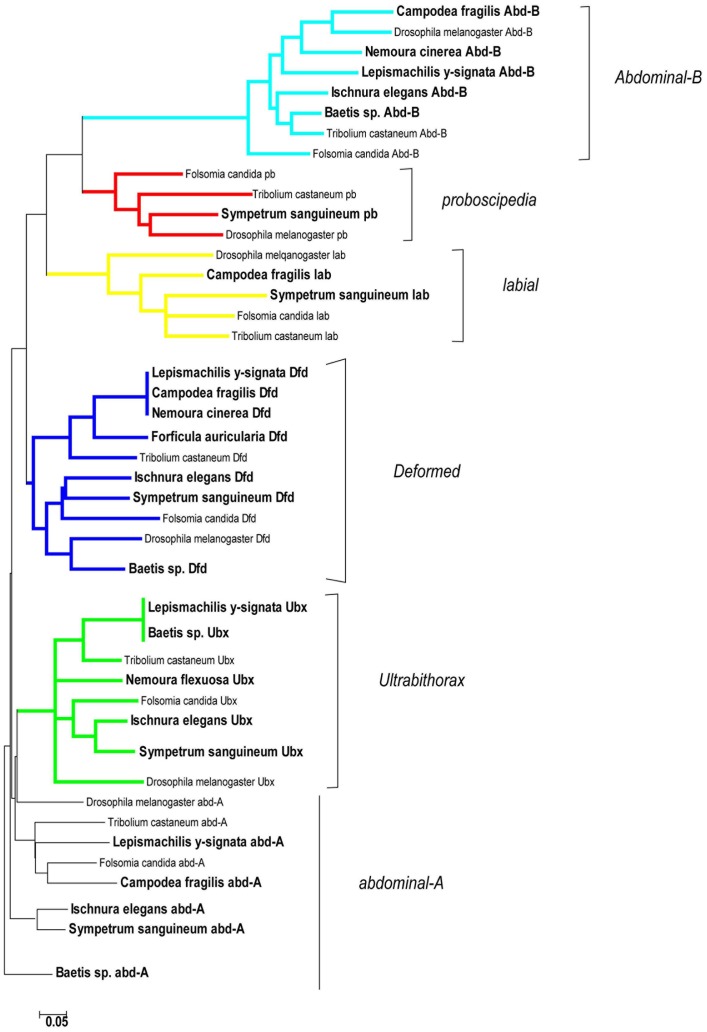
Phylogeny of insect orders. The phylogeny is based on information and figures in [56], [57], [58], [59]. Macro-evolutionary events in insect evolution, which are cited as being major Bauplan transitions, are mapped on the phylogeny. Pictures are modified after [60].

**Table 3:** Divergence rates of Hox genes.

			average P distance	Fossil calibration		Divergence rates		
group of taxa	nos. of taxa	nos. of sites	(%)	(million years)		(% per 10^6^ years)	Fossil record	Reference
**lab (Hox1)**								
Gastropoda	5	75	32	528	0.061		*Oelandiella (Latouchella) korobkovi*	Khomentovsky&Karlova, 1993
Cephalopoda	3	75	12	520	0.023		*Plectronoceras*	Dzik, 1981
Bivalvia	5	75	30.7	510	0.060		*Fordilla troyensis*	Pojeta et al., 1973
Crustacea	5	75	23.6	510	0.046		*Canadaspis sp.*	Briggs, 1978
Insecta	9	75	26	396	0.066		*Rhyniognatha hirsti*	Engel&Grimaldi, 2004
Mammalia Hox-A	6	75	15.3	195	0.078		*Hadrocodium wui*	Luo et al., 2001
Mammalia Hox-B	6	75	6.1	195	0.031		*Hadrocodium wui*	Luo et al., 2001
Mammalia Hox-D	5	75	9.5	195	0.049		*Hadrocodium wui*	Luo et al., 2001
**pb(Hox2)**								
Gastropoda	4	75	38.8	528	0.073		*Oelandiella (Latouchella) korobkovi*	Khomentovsky&Karlova, 1993
Bivalvia	3	75	29.3	510	0.057		*Fordilla troyensis*	Pojeta et al., 1973
Crustacea	6	75	27.1	510	0.053		*Canadaspis sp.*	Briggs, 1978
Insecta	7	75	27.4	396	0.069		*Rhyniognatha hirsti*	Engel&Grimaldi, 2004
Mammalia Hox-A	6	75	4.3	195	0.022		*Hadrocodium wui*	Luo et al., 2001
Mammalia Hox-B	6	75	7	195	0.036		*Hadrocodium wui*	Luo et al., 2001
**Dfd(Hox4)**								
Gastropoda	4	72	34.7	528	0.066		*Oelandiella (Latouchella) korobkovi*	Khomentovsky&Karlova, 1993
Bivalvia	3	72	26.1	510	0.051		*Fordilla troyensis*	Pojeta et al., 1973
Crustacea	7	72	26.9	510	0.053		*Canadaspis sp.*	Briggs, 1978
Insecta	14	72	28	396	0.071		*Rhyniognatha hirsti*	Engel&Grimaldi, 2004
Mammalia Hox-A	5	72	4.4	195	0.023		*Hadrocodium wui*	Luo et al., 2001
Mammalia Hox-B	6	72	4.6	195	0.024		*Hadrocodium wui*	Luo et al., 2001
Mammalia Hox-C	5	72	1	195	0.005		*Hadrocodium wui*	Luo et al., 2001
Mammalia Hox-D	5	72	3.7	195	0.019		*Hadrocodium wui*	Luo et al., 2001
**Scr(Hox5)**								
Gastropoda	3	76	23.1	528	0.044		*Oelandiella (Latouchella) korobkovi*	Khomentovsky&Karlova, 1993
Cephalopoda	4	76	25.1	520	0.048		*Plectronoceras*	Dzik, 1981
Bivalvia	3	76	21.8	510	0.043		*Fordilla troyensis*	Pojeta et al., 1973
Crustacea	6	76	28.2	510	0.055		*Canadaspis sp.*	Briggs, 1978
Insecta	15	76	24.5	396	0.062		*Rhyniognatha hirsti*	Engel&Grimaldi, 2004
Mammalia Hox-A	6	76	6.7	195	0.034		*Hadrocodium wui*	Luo et al., 2001
Mammalia Hox-B	5	76	1	195	0.005		*Hadrocodium wui*	Luo et al., 2001
Mammalia Hox-C	6	76	5.1	195	0.026		*Hadrocodium wui*	Luo et al., 2001
**Antp(Hox6)**								
Gastropoda	3	76	25	528	0.047		*Oelandiella (Latouchella) korobkovi*	Khomentovsky&Karlova, 1993
Cephalopoda	3	76	20	520	0.038		*Plectronoceras*	Dzik, 1981
Bivalvia	4	76	19.4	510	0.038		*Fordilla troyensis*	Pojeta et al., 1973
Crustacea	6	76	20	510	0.039		*Canadaspis sp.*	Briggs, 1978
Insecta	16	76	19.7	396	0.050		*Rhyniognatha hirsti*	Engel&Grimaldi, 2004
Mammalia Hox-A	4	76	8.3	195	0.043		*Hadrocodium wui*	Luo et al., 2001
Mammalia Hox-B	4	76	5.9	195	0.030		*Hadrocodium wui*	Luo et al., 2001
Mammalia Hox-C	4	76	11.1	195	0.057		*Hadrocodium wui*	Luo et al., 2001
**Ubx(Hox7)**								
Gastropoda	3	76	26.9	528	0.051		*Oelandiella (Latouchella) korobkovi*	Khomentovsky&Karlova, 1993
Cephalopoda	2	76	1.2	520	0.002		*Plectronoceras*	Dzik, 1981
Bivalvia	2	76	14	510	0.027		*Fordilla troyensis*	Pojeta et al., 1973
Crustacea	8	76	26.1	510	0.051		*Canadaspis sp.*	Briggs, 1978
Insecta	14	76	23.4	396	0.059		*Rhyniognatha hirsti*	Engel&Grimaldi, 2004
Mammalia Hox-A	5	76	5.3	195	0.027		*Hadrocodium wui*	Luo et al., 2001
Mammalia Hox-B	5	76	4.4	195	0.023		*Hadrocodium wui*	Luo et al., 2001
**abdA(Hox8)**								
Gastropoda	3	75	25.6	528	0.048		*Oelandiella (Latouchella) korobkovi*	Khomentovsky&Karlova, 1993
Cephalopoda	3	75	25.8	520	0.050		*Plectronoceras*	Dzik, 1981
Bivalvia	3	75	33.6	510	0.066		*Fordilla troyensis*	Pojeta et al., 1973
Crustacea	5	75	23.2	510	0.045		*Canadaspis sp.*	Briggs, 1978
Insecta	14	75	23.8	396	0.060		*Rhyniognatha hirsti*	Engel&Grimaldi, 2004
Mammalia Hox-B	5	75	4.5	195	0.023		*Hadrocodium wui*	Luo et al., 2002
Mammalia Hox-C	4	75	6.8	195	0.035		*Hadrocodium wui*	Luo et al., 2001
Mammalia Hox-D	4	75	8	195	0.041		*Hadrocodium wui*	Luo et al., 2001
**abdB(Hox11)**								
Gastropoda	2	75	28.6	528	0.054		*Oelandiella (Latouchella) korobkovi*	Khomentovsky&Karlova, 1993
Cephalopoda	2	75	15.9	520	0.031		*Plectronoceras*	Dzik, 1981
Crustacea	5	75	23.6	510	0.046		*Canadaspis sp.*	Briggs, 1978
Insecta	10	75	18.8	396	0.047		*Rhyniognatha hirsti*	Engel&Grimaldi, 2004
Mammalia Hox-A	4	75	7.5	195	0.038		*Hadrocodium wui*	Luo et al., 2001
Mammalia Hox-C	5	75	2.4	195	0.012		*Hadrocodium wui*	Luo et al., 2001
Mammalia Hox-D	5	75	3.3	195	0.017		*Hadrocodium wui*	Luo et al., 2001

The only Hox gene sequences previously isolated from Apterygota were from two orders, Thysanura and Collembola. The addition of 13 new sequences from Archaeognatha and Diplura doubles the number of apterygote insect orders with known Hox gene sequences. The Archaeognatha Hox gene sequences possibly present the best available roots for Hox genes in Hexapoda, allowing a reference point for estimations on the speed of sequence evolution of Hox genes in insects [34]. In general, the new data provide a starting point for phylogenetic and developmental studies investigating the apterygote-pterygote transition.

In the Pterygota Hox gene sequences have previously been known from the derived Hemiptera, Diptera, Hymenoptera, Orthoptera, Coleoptera (complete cluster) and Lepidoptera [21], [35], [36]. With 24 new sequences from Ephemeroptera, Odonata, Plecoptera, and Dermaptera we here add new sequences particularly from phylogenetically more basal insect orders (Table S1). These data are crucial for addressing the origin of pterygote insects, i.e. the invention and radiation of an insect bauplan armed with wings. Most recent molecular phylogenetic analyses suggest a basal position for Odonata within the Pterygota [37], making odonates particularly important for unraveling the evolutionary and developmental origin of insect wings [11], [19], [38]. We could isolate all 8 Hox genes for odonates as well as the homeotic gene Hox3 (*bicoid*). Only one other homeotic, but non-Hox gene, *ftz*, escaped our survey. Although we increased the number of pterygote insect orders with known Hox gene sequences from 6 to 10, there is still some 19 insect orders left for which no information on Hox gene sequences are available (see Table 2).

The main goal of our study was to add as many new Hox cluster gene sequences from phylogenetically particularly important insect orders to the database as possible. The primer pairs used in this study proved to be successful for all 10 Hox cluster genes, but they did not amplify all homeobox fragments from all insect orders investigated in this study. Filling these gaps will require a different approach and possibly different primer sets. In contrast to previously used degenerate Hox primers our newly designed “insect specific” primers amplify significantly larger fragments (to almost full length homeoboxes), 120–164 instead of some 80 bp [39], [40]. With respect to preparing the grounds for comparative studies on the evolution of the winged insect bauplan the genes *Scr*, *Antp* and *Ubx* are of immediate importance [5], [19], [41], [42], [43]. We have isolated fragments from all three genes from Archaeognatha, Ephemeroptera, Odonata, and Plecoptera. If Odonata should represent the most basal pterygote insects (see above) the new sequences from odonates will become indispensable for comparative studies on the evolution of Pterygota (Fig. 4 and Table S1).

### Insects Hox genes in Development and Evolution

From the very beginning of embryogenesis Hox genes control axes formation and the resulting body structuring in Bilateria (for controversial discussion on non-bilaterian animals see Kamm et al. [44]; Ryan et al. [45]; Schierwater et al. [16]; Schierwater and Kamm, [46] and refs. therein). Studies on model systems offered tremendous insights into the genetic principles of bilaterian development. Current EvoDevo research is urgently seeking comparative data from non-model animal systems, since most of the established model systems are phylogenetically quite derived. If one wants to unravel the invention of wings in insects for example, a key bauplan change that has fueled the unchallenged radiation success of pterygote insects, comparative data from the base of Pterygota are indispensable (Fig. 4). From higher pterygote insects we know that *Scr* and *Ubx* play key roles for the development of wings [19], [35], [42], [43], [47]–[49]. In the absence of comparative data from more basal pterygote insect orders, however, no conclusions on the role of *Scr* and *Ubx* for evolutionary origin of the insect wing can be drawn. The new sequences from several crucial insect orders provide a first step towards obtaining the missing data.

To what degree Hox genes can also directly contribute to phylogenetic analyses has been controversially discussed [28]. The genomic organization of Hox genes has supported several important clades at higher taxonomic levels [50], [51], [52]. At the sequence level of the homeobox or homeodomain one may also find phylogenetic signals at lower taxonomic levels [34], [53]. The main limitation though relates to the shortness of the sequence while the main strength arises from the unproblematic alignment [54].

### Insects Homeoboxes have Radiated Faster than Non-insect Homeobox Sequences

The addition of 37 new insect homeobox sequences allows to test the hypothesis that an increased radiation success correlates to an increased rate of sequence evolution in the regulatory Hox genes.

In 1965 Zuckerkandl and Pauling [55] suggested that mutations accumulate over time and that therefore the genetic divergence could be used to estimate the time of split between clades – the idea of the molecular clock was originated. We calculate the absolute rate of sequence evolution in the regulatory Hox genes. As fossils are the best estimates for the minimal age of a specific group, we have used the fossil records to estimate the sequence evolution rate (in % per million years) at which the different lineages have accumulated mutations in their Hox genes.

Comparison of p-distances within and between groups revealed a significant faster sequence evolution in the insects compared to other arthropods and mammals (Table 3). Average sequence evolution rate of Hox gene homeoboxes in insects is estimated as 0.06+/−0.003% per million years (mean +/− SE) and significantly higher than in non-insects (0.04+/−0.02; p<0.001 U-test, 2-sided).

To interpret these sequence evolution rates we have to keep in mind two important aspects. First, the Hox gene homeobox sequences available for the different groups do not necessarily reflect the overall diversity in this group. For Bivalvia, sequences are available from 5 out of the 10 recognized orders, for Cephalopda from 3 out of 11, for Gastropoda from 4 out of 23, for Maxillipoda from 5 out of 14 and for Insecta from 11 out of 32. In addition homeobox sequences are not always complete which can lead to an underestimation of the overall p-distance. Secondly, p-distances do not take substitution rate biases, differences in evolutionary rates among sites or multiple substitutions at the same site into account. All of the above can lead to an underestimation of sequence evolution rates. Nevertheless, the outstanding high sequence evolution rate in insects supports the hypothesis that the unchallenged radiation success of insects, and particularly flying insects, coincides with an increased sequence evolution rate in the most important regulatory genes for the a-p bauplan setup, i.e. the Hox genes. Based on former experiences with short homeobox fragments the probability for misclassification should be low [39], [40] and the latter should not contribute significantly to the observed high rates of sequence evolution in insects.

## Supporting Information

Figure S1 **Neighbor-Joining tree of all previously known** ***Scr*** **,** ***ftz*** **, and** ***Antp*** **sequences from those insect orders for which the complete set of Hox gene homeobox sequences is known:** ***Folsomia candida*** **(Colle),** ***Drosophila melanogaster*** **(Dipt) and** ***Tribolium castaneum*** **(Coleo).** Even the full length homeobox sequences allow no unambiguous grouping (see text).
(TIFF)

Table S1 **Taxa list with GenBank accession numbers and new generated sequences from this study.**
(XLSX)
