# Supplementary figures and images for: Isolation of Hox Cluster Genes from Insects Reveals an Accelerated Sequence Evolution Rate
Source: PLoS One. 2012 Jun 7;7(6):e34682. doi: 10.1371/journal.pone.0034682 (PMC3369913; doi:10.1371/journal.pone.0034682)

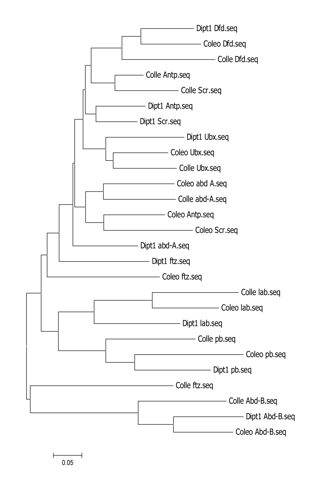

Supplement: Figure S1 — Neighbor-Joining tree of all previously known Scr , ftz , and Antp sequences from those insect orders for which the complete set of Hox gene homeobox sequences is known: Folsomia candida (Colle), Drosophila melanogaster (Dipt) and Tribolium castaneum (Coleo). Even the full length homeobox sequences allow no unambiguous grouping (see text). (TIFF) [file pone.0034682.s001.tif]
